# Expression and Binding Characteristics of AoraPBP3 in *Adoxophyes orana* (Lepidoptera: Tortricidae)

**DOI:** 10.3390/biology15141188

**Published:** 2026-07-18

**Authors:** Shaoqiu Ren, Hao Zeng, Weiwei Zhang, Zihan Yang, Xiulin Chen, Guangwei Li, Boliao Li

**Affiliations:** 1 Key Laboratory for Applied Ecology on Loess Plateau of Shaanxi Higher Education Institutions, College of Life Sciences, Yan’an University, Yan’an 716000, China; 17710451579@163.com (S.R.);; 2 Shaanxi Key Laboratory of Research and Utilization of Resource Plants on the Loess Plateau, Yan’an University, Yan’an 716000, China

**Keywords:** *Adoxophyes orana*, pheromone binding protein, binding property, molecular dynamics simulation

## Abstract

*Adoxophyes orana* is a major leafroller pest that damages fruit trees from Asia to Central Europe. The sex pheromone (SP) of *A. orana* comprises *Z*9-14:Ac, *Z*11-14:Ac, *Z*9-14:OH, *Z*11-14:OH, and *E*9-14: Ac. In this study, we characterized the ligand-binding properties of the AoraPBP3, combining experimental and computational approaches. RT-qPCR analyses demonstrated that *AoraPBP3* was highly expressed in antennae of both sexes, as well as male wings, and with transcript levels significantly higher in male antennae than in female. Fluorescence competitive binding assays (FCBAs) revealed that AoraPBP3 exhibited the highest binding affinity for *Z*11-14:Ac and no detectable affinity for *Z*11-14:OH. Meanwhile, AoraPBP3 bound to six host plant volatiles (HPVs), exhibiting a strong preference for pear ester. Molecular dynamics (MD) simulations evaluating AoraPBP3 interaction with the five SP components showed that the AoraPBP3–Z11-14:Ac complex possessed the lowest binding energy. Conversely, a high RMSD was observed in the AoraPBP3–*Z*11-14:OH complex, which might be attributed to too short distances between AoraPBP3 and *Z*11-14:OH throughout the simulation trajectory. By clarifying the functional and structural binding mechanisms of AoraPBP3 toward SP components and host plant recognition, this work will provide valuable insights for developing targeted mating disruption strategies to manage *A. orana* populations in orchards.

## Simple Summary

*Adoxophyes orana* is a major leafroller pest moth that damages fruit trees in Eurasia. Moth species rely on olfactory systems to locate host plants and mating partners. Sex pheromone of *A. orana* consists of *Z*9-14:Ac, *Z*11-14:Ac, *Z*9-14:OH, *Z*11-14:OH, and *E*9-14:Ac. OBPs transport the hydrophobic odor molecules through hydrophilic hemolymph to reach odorant receptors. A subclass of OBP, namely PBP, is currently a major focus. The precise mechanisms governing the ligand interactions of *A. orana* remain elusive. In this study, we conducted functional analyses of AoraPBP3 in both experimental and computational approaches. This protein displayed high binding affinity for *Z*11-14:Ac and no binding affinity for *Z*11-14:OH. Molecular dynamics (MD) simulations showed that the lowest total binding energy was found in the AoraPBP3–Z11-14:Ac complex, whereas the instability was found in the AoraPBP3–Z11-14:OH complex. These insights offer a foundation for developing novel pest management strategies that target and hinder mating behaviors.

## 1. Introduction

The summer fruit tortrix, *Adoxophyes orana*, is a highly polyphagous leafroller pest that impairs fruit trees spanning from Asia to central Europe [1,2,3,4]. This species infests more than 30 host plants, including apple, pear, peach, walnut, cherry, jujube, and mulberry [5,6]. The larvae primarily consume leaves and sometimes inflict superficial damage on fruit surfaces, particularly where fruits contact surrounding foliage. This external feeding scarifies the fruit epidermis, rendering it susceptible to secondary fungal infections that diminish fruit quality and marketability [2,6,7].

The sex pheromone (SP)-mediated communication system is critical for regulating the courtship and mating behaviors of most lepidopteran species [8]. For species in the Tortricidae family, early studies have shown that Tortricinae species typically use sex attractants with a 14-carbon chain, whereas Olethreutinae species employ attractants with a 12-carbon chain [9]. In the Olethreutinae subfamily, the SP of *Grapholita molesta* contains *Z*8-12:Ac, *E*8-12:Ac, *Z*8-12:OH, and 12:OH [10,11]. Its sibling species, *G. funebrana,* utilizes a pheromone bouquet consisting of *Z*8-12:Ac, *E*8-12:Ac, *Z*8-12:OH, *Z*8-14:Ac, and *Z*10-14:Ac [12]. The pheromone profile of *Cydia pomonella* comprises (*E*,*E*)-8,10-dodecadienol (codlemone), (*E*8,*Z*10)-12:OH, *E*9-12:OH, 12:OH, and 14:OH [13]. As for the Tortricinae subfamily, the SP of *Choristoneura fumiferana* is composed of *E*9-14:Ald and *Z*11-14:Ald in a ratio ranging from 95:5 to 97:3 [14,15,16], while that of *C. conflictana* features *Z*11-14:Ald, *E*11-14:Ald, and Z11-14:OH [17]. Furthermore, the SP of *Archips goyerana* consists of *Z*11-14:Ac, *E*11-14:Ac, *Z*9-14:Ac, and *Z*11-14:OH in an approximate ratio of 100:1.5:0.6:10 [18]. The SP of *A. orana* is characterized by a mixture of Z9-14:Ac, Z11-14:Ac, Z9-14:OH, Z11-14:OH, and *E*9-14:Ac [1,19].

The insect olfactory system is critical for locating host plants, seeking potential mates, and evading natural enemies [20]. Semiochemical molecules, most of which are hydrophobic, enter through numerous pores on the sensillar surface and penetrate the aqueous sensillar lymph. To evoke peripheral olfactory signaling, these lipophilic odor molecules must be transported across the hemolymph to the ORs. This process is mediated by soluble OBPs or CSPs [21,22]. Within Lepidoptera, OBPs are generally classified into PBPs, GOBPs, and ABPs [23]. Historically, PBPs were considered to function exclusively in SP recognition, while GOBPs were linked with the detection of host plant volatiles (HPVs) [24]. However, increasing evidence demonstrates that both PBPs and GOBPs are involved in the recognition and reception of SPs and HPVs [25,26,27,28]. The binding characteristics were well studied in several important fruit moth pests in the Olethreutinae subfamily, such as *G. molesta*, *G. funebrana*, and *C. pomonella*, whereas the binding mechanisms of PBP from Tortricinae species received less attention, especially using computational molecular docking and MD simulations.

A previous study confirmed that AoraGOBP2 strongly binds to Z9-14:Ac, Z9-14:OH, and Z11-14:OH, whereas AoraGOBP1 shows high binding affinity for Z9-14:Ac and several HPVs [29]. However, the functions of PBPs of this pest remain to be clarified. In the current study, we analyzed the expression pattern of AoraPBP3. Then, the binding affinity of AoraPBP3 was assessed for 5 SP components and 47 HPVs. Afterward, we conducted molecular docking and MD simulations to analyze interactions between AoraPBP3 and SP molecules. The results will help to understand the role of AoraPBP3 in olfaction and offer fresh perspectives on the pest control of *A. orana*.

## 2. Materials and Methods

### 2.1. Insect Rearing

The larvae of *A. orana* were collected from an apple orchard in Ganquan, Shaanxi Province, China (109°22′10″ E, 36°22′34″ N) in the summer of 2018 [6]. The population was successfully reared in the artificial climate box (BIC 300, Shanghai Boxun Medical Biological Instrument Co., Ltd., Shanghai, China) (Temperature: (25 ± 1) °C; relative humidity: (60 ± 10)%; photoperiod: L:D = 15 h:9 h). The larvae were reared on fresh mulberry leaves. The pupae were collected in 450 mL polyethylene cups. A female and a male moth were collected into a new polyethylene cup for mating and were fed on 5% sugar solution.

### 2.2. RNA Extraction, cDNA Synthesis, and RT-qPCR

Samples were prepared from about 100 pairs of antennae, heads or thorax of 10, abdomen from 5, legs from 25, and wings from 20 adults [29]. Total RNA was extracted using AG RNAex Pro Reagent (Accurate Biotechnology, Changsha, China). RNA quality was validated on a spectrophotometer (Allsheng, Hangzhou, China). The first-strand cDNA was synthesized according to the Evo M-MLV RT Kit with gDNA Clean for qPCR II (Accurate Biotechnology, Changsha, China). Polymerase chain reaction (PCR) was performed using 2 × Rapid Taq Master Mix (Vazyme, Nanjing, China) with the following conditions: 95 °C for 3 min; 35 cycles of 95 °C for 15 s, 55 °C for 15 s, and 72 °C for 20 s, and final extension at 72 °C for 5 min.

On a StepOnePlus™ Real-Time PCR System (ABI, Carlsbad, CA, USA), RT-qPCR was carried out using the 2 × Q3 SYBR qPCR Master Mix (TOLOBIO, Shanghai, China). Each 20 μL reaction mixture was composed of 10 μL of 2 × Q3 SYBR qPCR Master Mix, 0.4 μL of each primer (10 μM), 1 μL of diluted cDNA, and 8.2 μL of sterile water. The amplification protocol included 95 °C for 30 s, followed by 40 cycles of 95 °C for 10 s and 60 °C for 30 s. Three biological replicates were set for all developmental stages and tissues. Three technique replicates were applied for each biological sample. *EF1-α* and *β-actin* were chosen as reference genes. Relative expression level of *AoraPBP3* was calculated using the 2^−∆∆Ct^ method [30] and normalized to the geometric means of the two reference genes. Table S1.

### 2.3. Sequence Peptides Prediction and Alignment

The signal peptide of AoraPBP3 and PBP3 from other moths was predicted using SignalP 6.0 [31]. These sequences without signal peptides were aligned using MUSCLE (v. 3.8.1551) [32]. The alignment results were visualized using Jalview (v. 2.11.5.1) [33].

### 2.4. Prokaryotic Expression and Purification

The signal peptide-removed AoraPBP3 was cloned into the pET-28a vector using ClonExpress Ultra One Step Cloning Kit (Vazyme, Nanjing, China), with BamHI and HindIII restriction sites (Takara, Dalian, China). The resulting recombinant plasmid was transformed into *E. coli* BL21 (DE3) competent cells (TransGene, Beijing, China) for prokaryotic expression following a reported protocol [29]. The His-tagged target protein was purified by Ni-NTA agarose magnetic beads (7sea biotech, Shanghai, China), recovered in 20 mM Tris-HCl (pH 7.4) (Macklin, Shanghai, China) via dialysis, and subsequently used for in vitro FCBAs. Finally, protein purity was verified by SDS-PAGE, and the concentration of recombinant AoraPBP3 was assessed using a BCA protein assay kit (Vazyme, Nanjing, China).

### 2.5. Fluorescence Competitive Binding Assays

To evaluate the binding properties of AoraPBP3 to 5 SP components and 47 HPVs, FCBAs were performed on an F-2700 spectrophotofluorometer (Hitachi, Tokyo, Japan). To measure the affinity of the fluorescent probe 1-NPN, a 2 μM solution of AoraPBP3 in 20 mM Tris-HCl buffer (pH 7.4) was titrated with 1 mM methanol-dissolved 1-NPN (Sigma-Aldrich, Saint Louis, MO, USA) to achieve final concentrations from 2 to 20 μM. The fluorescence intensity was recorded using an excitation wavelength of 337 nm and an emission scan range of 370–550 nm. Titration continued until the increase in fluorescence intensity reached a plateau, and the *K_d_* of 1-NPN for AoraPBP3 was determined. For the competitive binding assays, a 2 mL protein solution containing 2 μM AoraPBP3 was titrated with each ligand at concentrations ranging from 2 to 18 μM. Fluorescence intensity was evaluated after a 2 min reaction period. The *K_i_* for each ligand was calculated using the formula *K_i_* = [*IC*_50_]/(1 + [1-NPN]/*K*_1-NPN_), where *IC*_50_ is the ligand concentration required to displace 50% of bound 1-NPN. Each experiment was performed in triplicate. Both *K_d_* and *K_i_* are presented as mean ± *S.E.M*.

### 2.6. 3D Modeling, Molecular Docking, and MD Simulation

The 3D structure of AoraPBP3 was predicted using AlphaFold2 (https://colab.research.google.com, accessed on 7 October 2025) [34,35]. The 3D structures of SP ligands were downloaded from PubChem (https://pubchem.ncbi.nlm.nih.gov, accessed on 17 June 2025) [36]. Molecular docking was performed using CB-Dock 2 (http://183.56.231.194:8001/cb-dock2/index.php, accessed on 13 October 2025) [37]. The interactions of AoraPBP3–ligand complexes were visualized by PyMol-open-source v. 3.1.0 [38].

The MD simulations were carried out using GROMACS (v. 2025.3) [39]. First, the TIP3P water model was specified in the GROMACS pdb2gmx step with the AMBER99SB force field [40]. The complex was neutralized by adding Na^+^. Then, the system energy was minimized using the steepest descent algorithm with a step size of 0.01 nm. System equilibration was performed in the NVT ensemble at 300 K for 1000 ps, followed by the NTP ensemble with the Parrinello–Rahman barostat for 2000 ps. After that, an 800 ns MD simulation was performed with an integration time step of 2 fs. Downstream analyses were carried out using GROMACS built-in tools (v. 2025.3) to calculate RMSD, RMSF, R_g_, and the centroid distance between the protein and the ligand [39]. Finally, the binding energies were calculated using gmx-MMPBSA (v. 1.6.5) [41].

### 2.7. Statistical Analysis

The results of RT-qPCR and FCBAs were statistically analyzed using GraphPad Prism (v. 6.0). One-way ANOVA followed by Tukey’s HSD test was performed to assess differences among multiple groups, with the threshold of *p* < 0.05. Student’s *t*-test was used to compare sex-biased differences (***: *p* < 0.001; **: *p* < 0.01; *: *p* < 0.05; ns: *p* ≥ 0.05).

## 3. Results

### 3.1. Sequence Analysis and Expression Profiles of AoraPBP3

**Figure 1:**
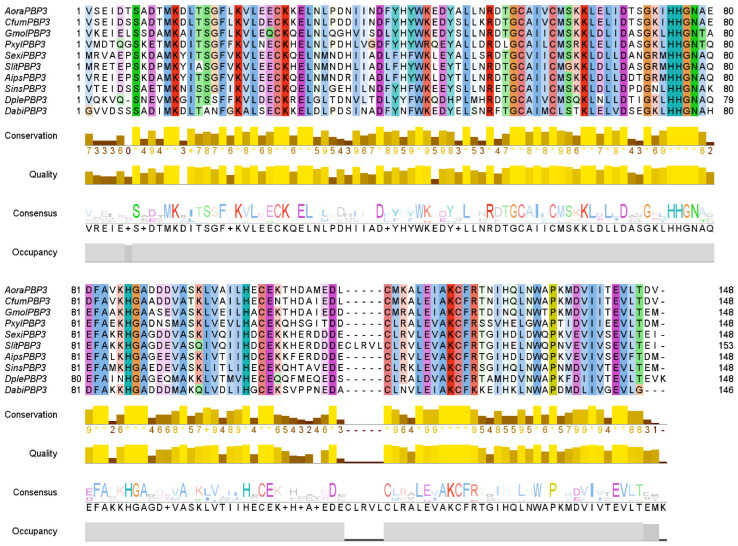
Alignment of AoraPBP3 and other PBP3 amino acid sequences. NCBI accession numbers: CfunPBP3 from *Choristoneura fumiferana*, XP_073954201.1. GmolPBP3 from *Grapholita molesta*, AHZ89399.1. PxylPBP3 from *Plutella xylostella*, QAT78217.1. AipsPBP3 from *Agrotis ipsilon*, AFM36758.1. SexiPBP3 from *Spodoptera exigua*, ACY78413.1. SlitPBP3 from *Spodoptera litura*, ACY78414.1. DabiPBP3 from *Dioryctria abietella*, AZK90260.1. SinsPBP3 from *Streltzoviella insularis*, QLI62029.1. DplePBP3 from *Danaus plexippus*, XP_032519595.1.

**Figure 2:**
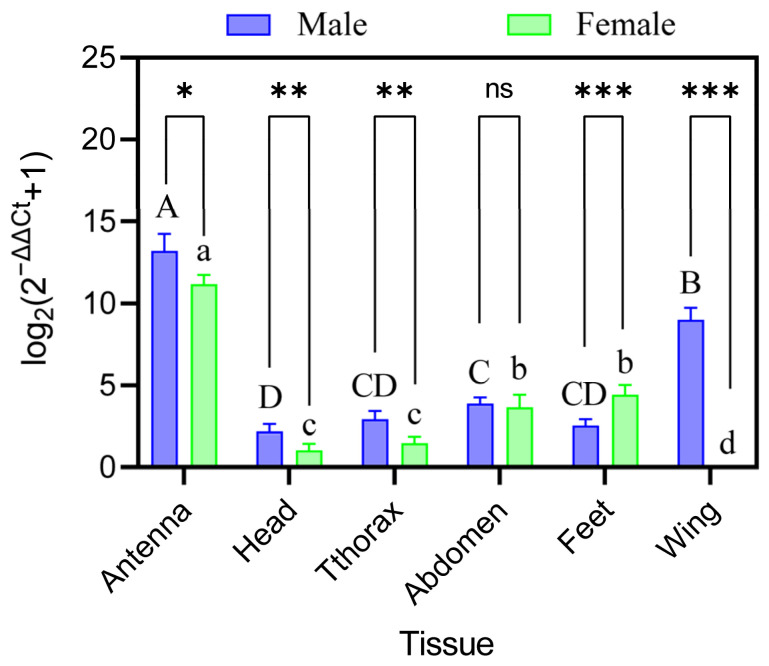
Relative expression of *AoraPBP3* in different adult tissues from *A. orana* using RT-qPCR. Different upper- and lower-case letters indicate significant differences among tissues using Tukey’s *post hoc* analyses after one-way ANOVA (*p* < 0.05). ns means no significant difference (*p* ≥ 0.05), and *, **, and *** mean significant differences (*p* < 0.05, *p* < 0.01, and *p* < 0.001, respectively) between sexes in the same tissue using the *t*-test. Error bar represents *S.E.M*.

The ORF of AoraPBP3 is 495 bp in length, encoding 164 amino acid residues characterized by six conserved cysteines (Figure 1). The predicted signal peptide of AoraPBP3 comprises 16 amino acids. The predicted molecular weight of mature AoraPBP3 was 16.73 kDa, and the isoelectric point was 4.78. The secondary structure of AoraPBP3 features seven α-helices: Glu3-Leu29 (α1), Asp33-His41 (α2), Arg52-Leu65 (α3), His76-His86 (α4), Asp90-His107 (α5), Leu113-Leu130 (α6), and Ile141-Asp147 (α7). Sequence alignment revealed that AoraPBP3 shared 59.15–93.92% identity with nine other lepidopteran PBP3 orthologs, exhibiting the highest amino acid sequence similarity (93.92%) with CfumPBP3 from *C. fumiferana* (Figure 1). RT-qPCR results indicated that *AoraPBP3* was abundant in antennae of both sexes and male wings (Figure 2).

### 3.2. Binding Characteristics of Recombinant AoraPBP3

The recombinant AoraPBP3 (rAoraPBP3) was successfully expressed in BL21 cells after IPTG induction (Figure S1). FCBAs showed that the *K_d_* of rAoraPBP3 for 1-NPN was 3.04 μM (95% CI: 2.69–3.43 μM), confirming 1-NPN as a suitable fluorescent probe for FCBAs (Figure S2). Then, we assessed the binding affinities of rAoraPBP3 towards five SP components and 47 HPVs. rAoraPBP3 exhibited the strongest binding affinity for *Z*9-14:Ac among five SP components, followed by *Z*11-14:Ac, and *E*9-14:Ac, alongside a slightly weaker affinity for Z9-14:OH, but with no binding affinity for *Z*11-14:OH (Table 1, Figure S2). Among the tested HPVs, rAoraPBP3 displayed the strongest binding affinity to pear ester, followed by a slightly weaker affinity for dibutyl phthalate, butyl butanoate, and α-farnesene (Table 1, Figure S3).

### 3.3. Structure Modeling, Molecular Docking, and MD Simulations

**Figure 3:**
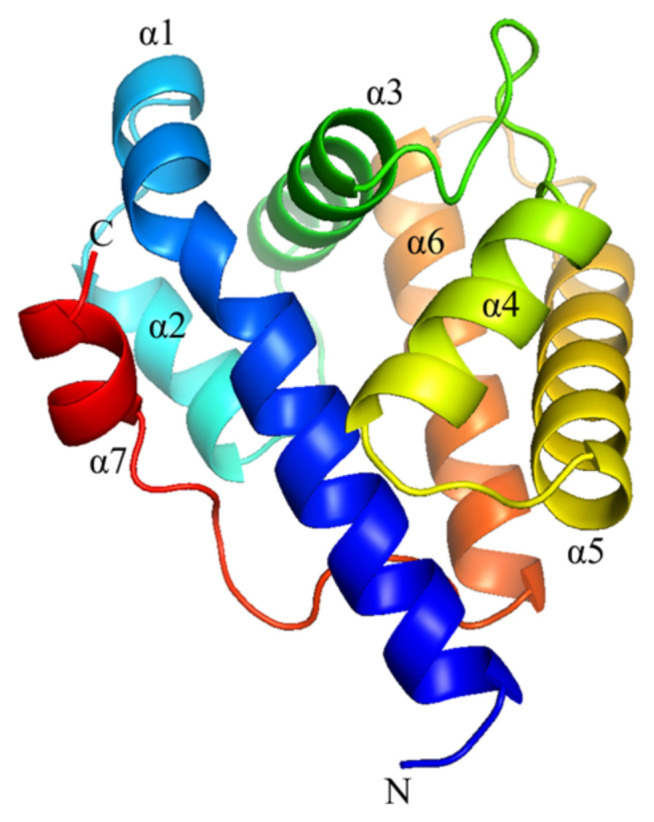
The 3D structure of AoraPBP3 based on alphafold3 modeling. **α1**–**7** indicates 1st to the 7th α-helices, which are colour-coded. **N** and **C** are the N-terminus and C-terminus, respectively.

**Figure 4:**
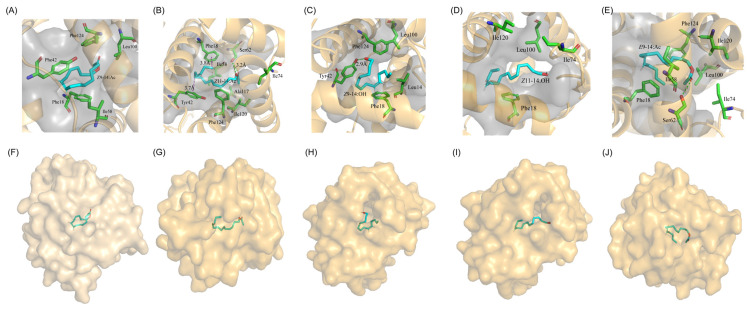
Interactions of AoraPBP3–ligand complex. (**A**,**F**) AoraPBP3–*Z*9-14:Ac. (**B**,**G**) AoraPBP3–*Z*11-14:Ac. (**C**,**H**) AoraPBP3–*Z*9-14:OH. (**D**,**I**) AoraPBP3–*Z*11-14:OH. (**E**,**J**) AoraPPB3–*E*9-14:Ac. (**A**–**E**), The purple and green dashed lines represent π-σ interactions and hydrogen bonds, respectively. The gray surface plot represents 5 Å around the ligand. The sticks indicate amino acid residues with the binding energy below −4.0 kJ/mol predicted based on MD simulations, or specific interactions predicted based on molecular docking. (**F**–**I**), The surface view of AoraPBP3–ligand complexes.

The predicted 3D model of AoraPBP3 revealed that AoraPBP3 had seven α-helices (α1–α7) (Figure 3). Ramachandran plots indicated the validity of the stereochemical and the high quality of the structure of this model (Figure S4). Subsequently, molecular docking results demonstrated that several hydrophobic residues form a cavity that mediates the binding interaction between AoraPBP3 and the SP molecules (Figure 4, Table S2).

**Figure 5:**
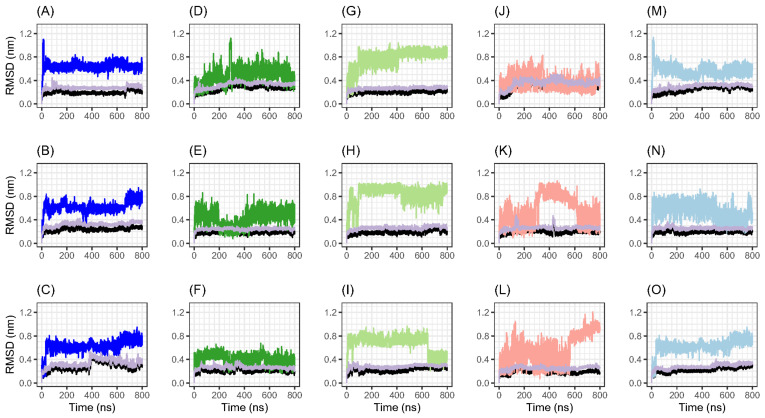
The root-mean-square deviation (RMSD) curves of AoraPBP3–ligand complexes. (**A**–**C**) AoraPBP3–*Z*9-14:Ac. (**D**–**F**) AoraPBP3–*Z*11-14:Ac. (**G**–**I**) AoraPBP3–*Z*9-14:OH. (**J**–**L**) AoraPBP3–*Z*11-14:OH. (**M**–**O**) AoraPPB3–*E*9-14:Ac. The black, altered-color, and light purple curves represent the RMSD of the protein, ligand, and complex, respectively.

**Figure 6:**
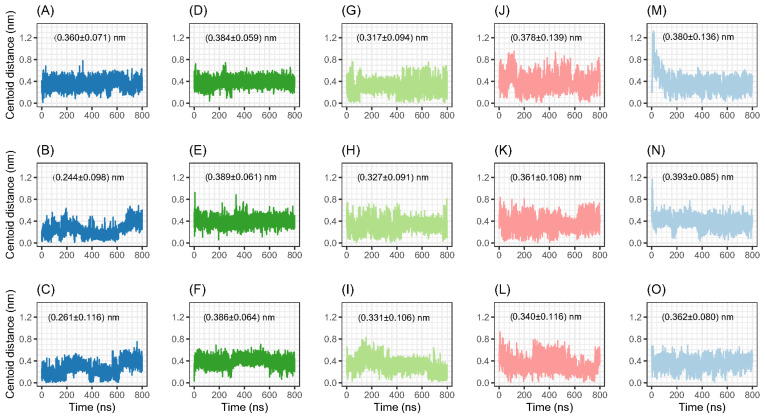
The centroid distance between AoraPBP3 and the sex pheromone ligands across an 800 ns MD simulation. (**A**–**C**) AoraPBP3–*Z*9-14:Ac. (**D**–**F**) AoraPBP3–*Z*11-14:Ac. (**G**–**I**) AoraPBP3–*Z*9-14:OH. (**J**–**L**) AoraPBP3–*Z*11-14:OH. (**M**–**O**) AoraPPB3–*E*9-14:Ac. The distances in the figure are presented as mean ± SD.

Three 800 ns MD simulations were carried out to evaluate the structural dynamics of AoraPBP3 in complex with each SP ligand. The MD simulation results showed that AoraPBP3 maintained overall structural stability, with RMSD values plateauing around 0.2 nm across all five systems. Regarding the ligands, the RMSD curves for Z9-14:Ac, Z11-14:Ac, and Z9-14:OH converged within the first 40 ns (Figure 5). Despite exhibiting an initial sharp shift, the RMSD trajectory of *E*9-14:Ac also achieved convergence after 300 ns. However, the RMSD curve of *Z*11-14:OH abruptly fluctuated from 300 ns to 600 ns in the second repetition and after 560 ns in the third repetition, suggesting a highly unstable binding state within the AoraPBP3 pocket (Figure 5). In addition, the modeled AoraPBP3 maintained a highly compact conformation throughout the simulation, as evidenced by an average radius of gyration (R_g_) of around 1.5 nm across all five complexes (Figure S5). This instability was further supported by the centroid distance curves between the SP ligands and the AoraPBP3 binding pocket. These measurements indicated that while the AoraPBP3–*Z*11-14:Ac and AoraPBP3–*Z*9-14:OH complexes remained stable throughout the entire 800 ns trajectory, the AoraPBP3–*Z*9-14:Ac and AoraPBP3–*E*9-14:Ac complexes stabilized in the majority of MD simulations, and the AoraPBP3–*Z*11–14:OH complex experienced distinct MD simulations among three repetitions (Figure 6).

**Figure 7:**
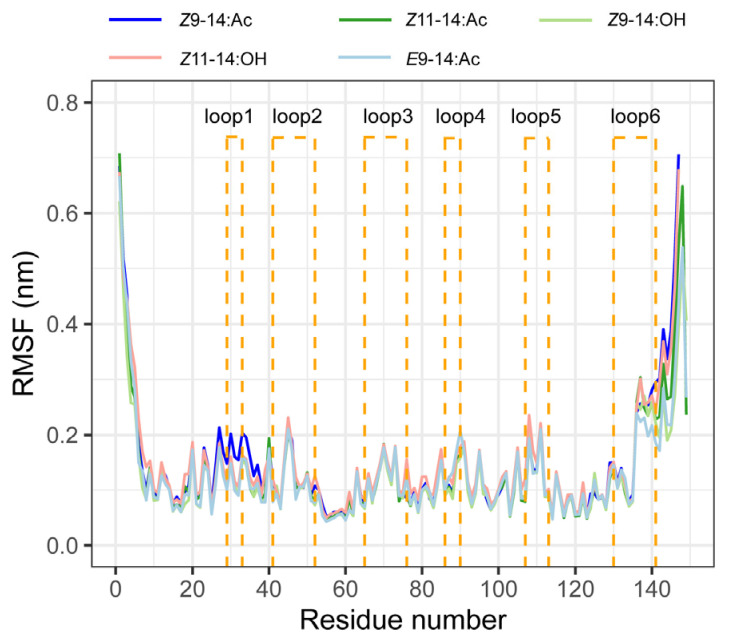
The RMSF curves of AoraPBP3–ligand complexes. The RMSF value of each complex indicates the average of three replicates. The orange dashed boxes indicate amino acid residues that consist of loops.

The RMSF values in most structural regions remained below 0.2 nm in these five complexes (Figure 7). Pronounced structural mobility was observed in the N-terminal regions (approximately < 8 aa) and the C-terminal regions (approximately > 138 aa), where RMSF values exceeded 0.2 nm. Notably, the local fluctuations within loop 1, loop 5, and loop 6 showed slight differences among the five complexes, partly corresponding to the various binding affinities of AoraPBP3 towards the five SP components (Figure 7).

### 3.4. Binding Free Energy Calculation and Per-Residue Energy Decomposition

Based on the trajectory sampled during an 800 ns MD simulation, the Δ*G_bind_* of five complexes was calculated. As shown in Table 2, van der Waals energy (Δ*G_vdW_*) occupied the majority of the binding energy for all five complexes. Δ*G_vdW_* of AoraPBP3 binding to three esters was lower than that of two alcohols. The Δ*G_vdW_* of AoraPBP3–*Z*11-14:Ac was the lowest among these five complexes.

**Figure 8:**
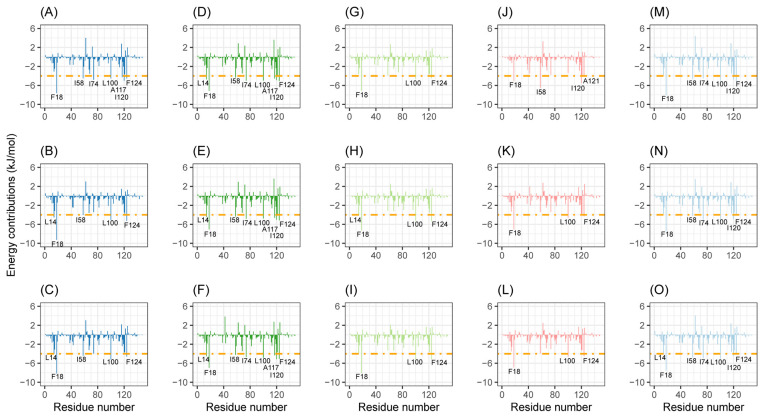
The per-residue free energy decomposition of AoraPBP3–ligand complexes. Dashed orange lines indicate total binding energy below −4.00 kJ/mol. (**A**–**C**) AoraPBP3–*Z*9-14:Ac. (**D**–**F**) AoraPBP3–*Z*11-14:Ac. (**G**–**I**) AoraPBP3–*Z*9-14:OH. (**J**–**L**) AoraPBP3–*Z*11-14:OH. (**M**–**O**) AoraPPB3–*E*9-14:Ac.

Per-residue free-energy decomposition analyses (Figure 8) showed that Phe18 exhibited the lowest binding free energy, providing the largest energetic contribution among residues across five complexes among all the residues. Additionally, Phe124 and Leu100 contributed significantly to ligand stabilization in all five complexes, except in a repetition of AoraPBP3–*Z*11-14:OH. Ile58 contributed substantially to the binding of AoraPBP3 to the acetate components (*Z*9-14:Ac, *Z*11-14:Ac, and *E*9-14:Ac), but not to the stabilization of the alcohol ligands (*Z*9-14:OH and *Z*11-14:OH) (Figure 8). Interestingly, Ala117 and Ile120 specifically made significant favorable contributions to the binding of *Z*11-14:Ac, and Ile120 contributed to the binding of *E*9-14:Ac, highlighting their potential role in ligand selectivity (Figure 8).

## 4. Discussion

In the tissue expression pattern of *A. orana* adults, *AoraPBP3* was predominantly expressed in antennae of both sexes, as well as male wings (Figure 2). This finding is consistent with *PBP3s* in several other moth species, which were predominantly expressed in antennae, and higher amounts in male antennae than in female [26,42,43,44]. Conversely, no sex-biased expression of PBP3 was observed in the other species, such as in *Spodoptera litura* [25] and *G. funebrana* [45]. It is noteworthy that the relatively high level of *AoraPBP3* was found in male wings. Although not common, *PBP* was also expressed at relatively high levels in several other moth species, including *MvitPBP1*, *MvitPBP2*, and *MvitPBP3* in female *Maruca vitrata* [46], and *CsasPBP1* and *CsasPBP3* in female *C. sasakii* [26]. We could not determine whether the high level of *PBP* genes in male wings is common in Tortricinae species, and the rich amount of AoraPBP3 in male wings warrens further validates using immunocytochemical localization. These results likely indicate functional disparity among PBP3 orthologs across various branches.

The results of FCBAs indicated that AoraPBP3 possessed relatively high binding affinities for *Z*11-14:Ac, Z9-14:Ac, and *E*9-14:Ac, a marginally weaker affinity for *Z*9-14:OH, and no detectable affinity for Z11-14:OH (Table 1). PBP3 orthologs across diverse lepidopteron species exhibited various binding spectra. HarmPBP3 from *Helicoverpa armigera* and HassPBP3 from *H. assulta* displayed high binding affinities for six SP components, with a marked preference for two ester components [47]. AipsPBP3 from *Agrotis ipsilon* exhibited its highest binding affinity for Z11-16:Ac, while retaining the capacity to bind the other four SP components [42]. OachPBP3 from *Orthaga achatina* bound to three secondary SP components but failed to recognize the primary component *Z*11-16:Ac [48]. TabsPBP3 from *Tuta absoluta* displayed a stronger affinity for (3*E*,8*Z*)-14:Ac, the secondary SP component, than (3*E*,8*Z*,11*Z*)-14:Ac, the primary SP component [49]. In *Peridroma saucia*, PsauPBP3 exhibited high affinities for Z11-16:Ac and Z9-14:Ac, alongside minor affinities for *Z*9-16:Ac, *Z*11-16:OH, *Z*11-16:Ald, and *Z*9-16:OH [44]. CsasPBP3 from *Carposina sasakii* exhibited a specific affinity towards Z7-11One:20C than Z7-11One:19C [26]. As for *G. funebrana*, GfunPBP3 exhibited relatively strong binding affinities for *E*8-12:Ac, *Z*8-12:Ac, and *Z*8-12:OH, coupled with moderate affinities for *Z*8-14:Ac and *Z*10-14:Ac [45]. However, PBP3 from *Sesamia inference* [50] and *G. molesta* [51] showed no obvious binding affinities for their respective SP components. In the current study, AoraPBP3 from *A. orana*, in alignment with many other lepidopteran species, plays a significant physiological role in the peripheral recognition and transport of SP components. AoraPBP3 showed the highest sequence similarity with CfumPBP3 from *C. fumiferana* among ten PBP3 orthologs (Figure 1). Since the sequence of CfumPBP3 was published in 2000 [52], the function of this protein and other PBP3 orthologs in Tortricinae species is expected to be illuminated.

An increasing number of studies have shown that the function of PBPs is not limited to binding SP components but also extends to binding HPVs. For instance, CsasPBP3 from *C. sasakii* has the binding affinities for (*Z*,*E*)-α-farnesene, hexaldehyde, and nonanal [26]. TabsOBP2 from *Tuta absoluta* exhibited relatively strong binding affinities to several ternenoid and green leaf volatile components [53]. EsigPBP3 from *Endoclita signifier* showed high binding affinities for α-terpinene, (*E*)- β-ocimene, α-pinene, and β-pinene [54]. In the present study, AoraPBP3 also exhibited relatively strong binding affinities to HPVs (Table 1), as supported by results from the above lepidopteran species.

MD simulation constitutes a powerful methodology for investigating protein–ligand interactions. In the current study, MD analyses revealed that AoraPBP3 formed stable complexes with Z9–14:Ac, Z11–14:Ac, *E*9–14:Ac, and Z9–14:OH, as evidenced by RMSD curves (Figure 5). On the contrary, the RMSD curves of three AoraPBP3–*Z*11-14:OH complexes were quite unstable and divergent (Figure 5J–L). The RMSD values were high from 300 ns to 600 ns in the second repetition and after 560 ns in the third repetition (Figure 5K–L). Interestingly, the centroid distances between AoraPBP3 and *Z*11-14:OH were relatively shorter than those of the other MD simulation periods (Figure 6), which likely induced stronger repulsive or unfavorable negative interactions. Based on the MD simulation results, the Δ*G_bind_* value was lowest in AoraPBP3–*Z*11-14:Ac and highest in AoraPBP3–*Z*11-14:OH (Table 2), parallel to the lowest *K_i_* of AoraPBP3 binding to *Z*11-14:Ac and the highest *K_i_* to *Z*11-14:OH calculated from the FCBAs (Table 1). In *Plutella xylostella*, the *K_i_* value of PxylPBP3 was 0.64 ± 0.13 μM for *Z*11-16:Al and 0.21 ± 0.05 μM for *Z*11-16:Ac based on FCBAs, whereas the Δ*G_bind-alchem_* was −23.82 ± 0.86 kcal/mol for *Z*11-16:Al and −29.65 ± 0.90 kcal/mol for *Z*11-16:Ac [55]. Although limited studies have involved the direct comparison of PBP3s between FCBAs and MD simulations, the results of MD simulations could robustly support the results of FCBAs.

Per-residue free-energy decomposition analysis is a useful approach for identifying the key amino acid residues that interact with ligands. Phe34, Tyr37, Trp38, and Arg111 were identified as key residues mediating the binding of PxylPBP3 from *Plutella xylostella* to Z11-16:Ac [55]. Thr10, Phe13, Ile53, Ile95, and Phe119 were predicted to be critical residues of PsauPBP3 from *P. saucia* that interacted with *Z*9-14:Ac and *Z*11-16:Ac [44]. In the present study of *A. orana*, Phe18, Ile120, and Phe124 were designated as key residues that interact across all five SP components. Ile58 contributed substantially to the three ester SPs but not to the two alcohol SPs (Figure 8). Additionally, Ala117 and Ile120 acted synergistically with Phe124 to enhance binding affinity toward *Z*11-14:Ac (Figure 8), potentially explaining why *Z*11-14:Ac served as the most energetically favorable ligand for AoraPBP3. The predicted key amino acids need to be verified for site-directed mutagenesis validation in the future.

## 5. Conclusions

In summary, *AoraPBP3* was abundantly expressed in antennae of both sexes and male wings. FCBAs revealed that AoraPBP3 exhibited the highest binding affinity toward *Z*11-14:Ac, followed by *Z*9-14:Ac, *E*9-14:Ac, and *Z*9-14:OH, and no binding affinity to *Z*11-14:OH. Among the HPVs, AoraPBP3 displayed preferential binding toward pear ester. Furthermore, MD simulations identified that Phe18, Ile120, and Phe124 were critical residues driving these pheromone interactions. Taken together, these insights elucidate the structural mechanisms of PBP3 in moth species, assisting in optimizing and enhancing pheromone-based pest control strategies.

## Figures and Tables

**Table 1:** Binding affinities of AoraPBP3 to sex pheromones and host plant volatiles.

Ligand Name	CAS Number	*IC*_50_ (μM)	*K_i_* (μM)	Whether the Fluorescence Value Decreased 50%
Z9-14:Ac	16725-53-4	14.82 ± 0.38	8.49 ± 0.07	Yes
*Z*11-14:Ac	20711-10-8	12.90 ± 0.10	7.81 ± 0.06	Yes
*Z*9-14:OH	35153-15-2	14.92 ± 0.05	9.02 ± 0.01	Yes
*Z*11-14:OH	34010-15-6	23.59 ± 0.90	14.48 ± 0.60	No
*E*9-14:Ac	23192-82-7	14.33 ± 0.47	8.84 ± 0.15	Yes
1-Hexanol	111-27-3	69.18 ± 1.56	41.78 ± 0.94	No
Benzyl alcohol	100-51-6	37.88 ± 0.76	22.88 ± 0.46	No
(Z)-3-hexen-1-ol	928-96-1	30.95 ± 0.93	18.69 ± 0.56	No
Farnesol	4602-84-0	24.27 ± 0.04	14.66 ± 0.02	No
Nerolidol	3790-78-1	24.28 ± 0.04	14.67 ± 0.04	No
1-Penten-3-ol	616-25-1	46.18 ± 0.27	27.89 ± 0.16	No
Linalool	78-70-6	45.07 ± 1.04	27.22 ± 0.63	No
1-Heptanol	111-70-6	59.03 ± 1.33	35.65 ± 0.80	No
1-Decanol	112-30-1	25.90 ± 0.62	15.64 ± 0.38	No
1-Dodecanol	88170-32-5	55.72 ± 2.10	33.65 ± 1.27	No
Cineole	470-82-6	45.45 ± 0.43	27.45 ± 0.26	No
(*Z*,*E*)-phytol	7541-49-3	16.47 ± 0.35	9.95 ± 0.21	Yes
Isooctyl alcohol	26952-21-6	44.20 ± 0.27	26.70 ± 0.16	No
Butyl butanoate	109-21-7	11.07 ± 0.12	6.68 ± 0.07	Yes
Methyl salicylate	119-36-8	24.18 ± 0.48	14.59 ± 0.29	No
Ethyl acetate	141-78-6	27.01 ± 0.26	16.30 ± 0.14	No
Ethyl valerate	539-82-2	42.00 ± 0.33	25.34 ± 0.21	No
Dibutyl phthalate	84-74-2	10.51 ± 0.23	6.34 ± 0.14	Yes
Ethyl isovalerate	108-64-5	49.96 ± 0.36	30.14 ± 0.19	No
cis-3-Hexenyl butyrate	16491-36-4	54.96 ± 0.94	33.16 ± 0.33	No
Butyl acetate	123-86-4	33.16 ± 0.83	20.01 ± 0.50	No
Ethyl butyrate	105-54-4	37.34 ± 0.36	22.53 ± 0.22	No
Pear ester	3025-30-7	6.00 ± 0.06	3.62 ± 0.03	Yes
Ethyl tiglate	5837-78-5	18.7 ± 0.23	11.28 ± 0.24	No
Isoamyl acetate	123-92-2	28.77 ± 0.59	17.36 ± 0.35	No
Methyl palmitate	112-39-0	33.25 ± 0.46	20.07 ± 0.27	No
Butyl propanoate	590-01-2	24.94 ± 1.01	15.05 ± 0.61	No
(Z)-3-Hexen-1-yl 3-methylbutanoate	35154-45-1	29.11 ± 0.27	17.57 ± 0.l8	No
cis-3-hexenyl acetate	1708-82-3	19.11 ± 0.23	11.53 ± 0.14	No
Hexanal	66-25-1	30.47 ± 0.22	18.38 ± 0.14	No
Heptanal	111-71-7	22.64 ± 0.15	13.67 ± 0.09	No
Nonanal	124-19-6	74.07 ± 4.91	44.69 ± 2.95	No
Decanal	112-31-2	29.12 ± 0.28	17.57 ± 0.18	No
Benzaldehyde	100-52-7	59.51 ± 1.87	35.91 ± 1.15	No
trans-2-Hexenal	6728-26-3	79.30 ± 2.47	47.85 ± 1.53	No
Citral	5392-40-5	47.02 ± 0.48	28.37 ± 0.29	No
Isobutyraldehyde	78-84-2	32.11 ± 0.23	19.38 ± 0.09	No
α-Farnesene	502-61-4	13.04 ± 0.08	7.87 ± 0.05	Yes
Camphene	79-92-5	35.60 ± 0.47	21.48 ± 0.28	No
α-Ocimene	502-99-8	15.74 ± 0.12	9.50 ± 0.07	Yes
α-Pinene	7785-70-8	52.83 ± 1.79	31.88 ± 1.10	No
β-Caryophyllene	87-44-5	2628 ± 243.7	1586 ± 145.5	No
3-Carene	13466-78-9	24.39 ± 0.18	14.70 ± 0.09	No
2-methylprop-1-enylbenzene	768-49-0	30.21 ± 1.18	18.23 ± 0.69	No
α-Phellandrene	99-83-2	23.22 ± 0.24	14.01 ± 0.15	No
3,7-Dimethyl-2,6-octadienenitrile	5146-66-7	21.28 ± 0.31	12.96 ± 0.06	No
Benzonitrile	100-47-0	24.20 ± 0.57	14.83 ± 0.15	No

**Table 2:** Binding free energy for the AoraPBP3–ligand complexes (kJ/mol) (mean ± SD).

Complex	Electrostatic Energy (∆*G_ele_*)	van der Waals Energy (∆*G_vdw_*)	Polar Solvation Energy (∆*G_PB_*)	Nonpolar Solvation Energy (∆*G_SA_*)	∆*H*	−*T*∆*S*	∆*G_bind_*	Average ∆*G_bind_*
AoraPBP3– *Z*9-14:Ac	−8.505 ± 6.545	−196.727 ± 10.664	66.508 ± 10.198	−26.366 ± 0.881	−165.677 ± 12.595	25.265	−140.421	−141.658 ± 1.072 a
	−10.835 ± 5.601	−196.129 ± 10.579	60.531 ± 7.595	−24.988 ± 1.190	−173.142 ± 12.155	30.897	−142.245	
	−8.414 ± 6.252	−193.192 ± 10.049	58.624 ± 9.208	−25.794 ± 1.074	−168.776 ± 10.759	26.466	−142.309	
AoraPBP3– *Z*11-14:Ac	−8.339 ± 5.207	−198.044 ± 9.368	61.133 ± 8.520	−26.385 ± 0.765	−171.635 ± 11.815	18.866	−152.769	−151.418 ± 3.383 a
	−8.666 ± 5.381	−198.471 ± 8.781	62.287 ± 7.723	−26.425 ± 0.736	−171.314 ± 11.023	17.397	−153.917	
	−7.609 ± 5.952	−196.268 ± 9.306	67.254 ± 10.605	−26.481 ± 0.771	−163.103 ± 12.428	15.534	−147.569	
AoraPBP3– *Z*9-14:OH	−10.409 ± 5.782	−162.736 ± 8.374	59.052 ± 8.802	−23.291 ± 0.898	−137.384 ± 9.900	11.985	−125.399	−122.329 ± 6.785 b
	−12.514 ± 4.589	−163.031 ± 9.124	58.464 ± 7.692	−22.511 ± 0.895	−139.592 ± 10.567	25.042	−114.551	
	−10.755 ± 4.872	−162.575 ± 8.500	53.401 ± 5.311	−22.420 ± 0.789	−142.349 ± 9.113	15.313	−127.036	
AoraPBP3– *Z*11-14:OH	−12.215 ± 7.981	−173.656 ± 10.373	63.009 ± 8.851	−23.713 ± 0.694	−146.575 ± 11.910	21.266	−125.309	−116.382 ± 7.961 b
	−11.389 ± 6.557	−164.430 ± 9.933	61.509 ± 9.909	−23.239 ± 0.939	−137.548 ± 11.718	23.729	−113.819	
	−11.293 ± 6.350	−163.875 ± 9.457	57.579 ± 8.643	−23.063 ± 1.073	−140.652 ± 10.417	30.633	−110.019	
AoraPBP3– *E*9-14:Ac	−9.819 ± 6.850	−193.739 ± 9.840	60.474 ± 9.646	−25.854 ± 1.131	−168.938 ± 11.609	20.568	−148.370	−149.226 ± 2.986 a
	−8.165 ± 5.957	−194.786 ± 9.936	63.342 ± 9.703	−26.654 ± 0.912	−166.265 ± 11.827	19.298	−146.966	
	−9.676 ± 6.401	−193.307 ± 9.752	60.422 ± 9.352	−25.918 ± 1.076	−168.478 ± 11.498	15.783	−152.695	

∆*G_bind_* = ∆*H* − *T*∆*S*, and ∆*H* = ∆*G_ele_* + ∆*G_vdw_* + ∆*G_PB_* + ∆*G_SA_*. ∆*H* represents enthalpy change, whereas *T*∆*S* indicates the absolute temperature multiply the entropy change. The letters in the last column represent significant differences in ∆*G_bind_* among five complexes using one-way ANOVA and Tukey’s tests.

## Data Availability

Data inquiries can be directed to the corresponding author.

## Supplementary Materials

The following supporting information can be downloaded at: https://www.mdpi.com/article/10.3390/biology15141188/s1, Figure S1: SDS-PAGE analysis of recombinant pET-28a/AoraPBP3; Figure S2: Binding curve of AoraPBP3 to 1-NPN and relative Scatchard plot analysis; Figure S3: Competitive binding curves of AoraPBP3 to various ligands; Figure S4: Ramachandran plots of AoraPBP3 model; Figure S5: Radius of gyration (R_g_) dynamics in the AoraPBP3–Ligand complexes; Table S1: Primers used for RT-qPCR and prokaryotic expression; Table S2: Amino acids within 5 Å around sex pheromone molecules based on molecular docking.

## Abbreviations

The following abbreviations are used in this manuscript:

SP	Sex pheromone
*Z*8-12:Ac	(*Z*)-8-dodecenyl acetate
*E*8-12:Ac	(*E*)-8-dodecenyl acetate
*Z*8-12:OH	(*Z*)-8-dodecen-1-ol
12:OH	dodecanol
*Z*8-14:Ac	(*Z*)-8-tetradecenyl acetate
*Z*10-14:Ac	(*Z*)-10-tetradecenyl acetate
(*E*8,*Z*10)-12:OH	(*E*,*Z*)-8,10-dodecadienol
*E*9-12:OH	(*E*)-9-dodecenol
14:OH	tetradecanol
*E*9-14:Ald	(*E*)-9-tetradecenal
*Z*11-14:Ald	(*Z*)-11-tetradecenal
*E*11-14:Ald	(*E*)-11-tetradecenal
*Z*9-14:Ac	(*Z*)-9-tetradecenyl acetate
*Z*11-14:Ac	(*Z*)-11-tetradecenyl acetate
Z9-14:OH	(*Z*)-9-tetradecen-1-ol
*Z*11-14:OH	(*Z*)-11-tetradecen-1-ol
*E*11-14:Ac	(*E*)-11-tetradecenyl acetate
(3*E*,8*Z*)-14:Ac	(3*E*,8*Z*)-tetradecadien-1-yl acetate
(3*E*,8*Z*,11*Z*)-14:Ac	(3*E*,8*Z*,11*Z*)-tetradecatrien-1-yl acetate
*Z*7-11One:20C	*Z*-7-eicosene-11-one
*Z*7-11One:19C	*Z*-7-nonadecene-11-one
OR	olfactory receptor
OBP	odorant-binding protein
CSP	chemosensory protein
PBP	pheromone-binding protein
GOBP	general odorant-binding protein
ABP	antennae binding protein
FCBA	fluorescence competitive binding assay
1-NPN	N-phenyl-1-naphthylamine
*K_d_*	binding dissociation constant
*K_i_*	inhibition constant
3D modeling	three-dimensional modeling
MD simulations	molecular dynamics simulations
NVP ensemble	number of particles-volume-temperature ensemble
NPT ensemble	number of particles-pressure-temperature ensemble
RMFD	root mean square deviation
RMSF	root mean square fluctuation
R_g_	radius of gyration
one-way ANOVA	one-way analysis of variance
RT-qPCR	real-time quantitative polymerase chain reaction
