# Supplementary material for: Expression and Binding Characteristics of AoraPBP3 in Adoxophyes orana (Lepidoptera: Tortricidae)
Source: Biology (Basel). 2026 Jul 18;15(14):1188. doi: 10.3390/biology15141188 (PMC13405892; doi:10.3390/biology15141188)
Supplement: Supplementary file 1 [file biology-15-01188-s001.zip › biology-4409206-supplementary.pdf]

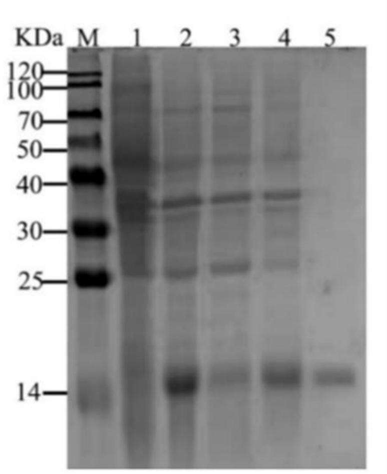

Figure S1. SDS-PAGE analysis of recombinant AoraPBP3. Lane M, protein molecular weight marker; Lane 1 and 2, before and after IPTG-induced pET28a(+)-AoraPBP3; Lane 3, supernatant of IPTG-induced pET28a(+)-AoraPBP3; Lane 4, inclusion bodies of IPTGinduced pET28a(+)-AoraPBP3. Lane 5, purified rAoraPBP3.

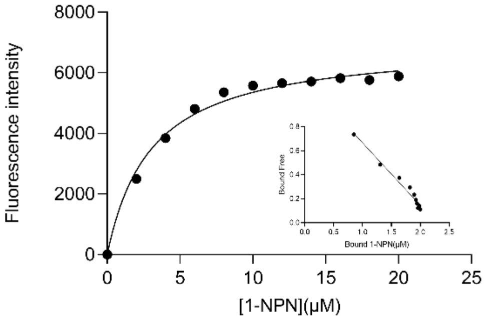

Figure S2. Binding curves and Scatchard plots (insert) of the fluorescence probe 1-NPN to AoraPBP3. The binding curve and the relative Scathard plot indicate the binding constants of AoraPBP3/1-NPN complex was 3.04 uM.

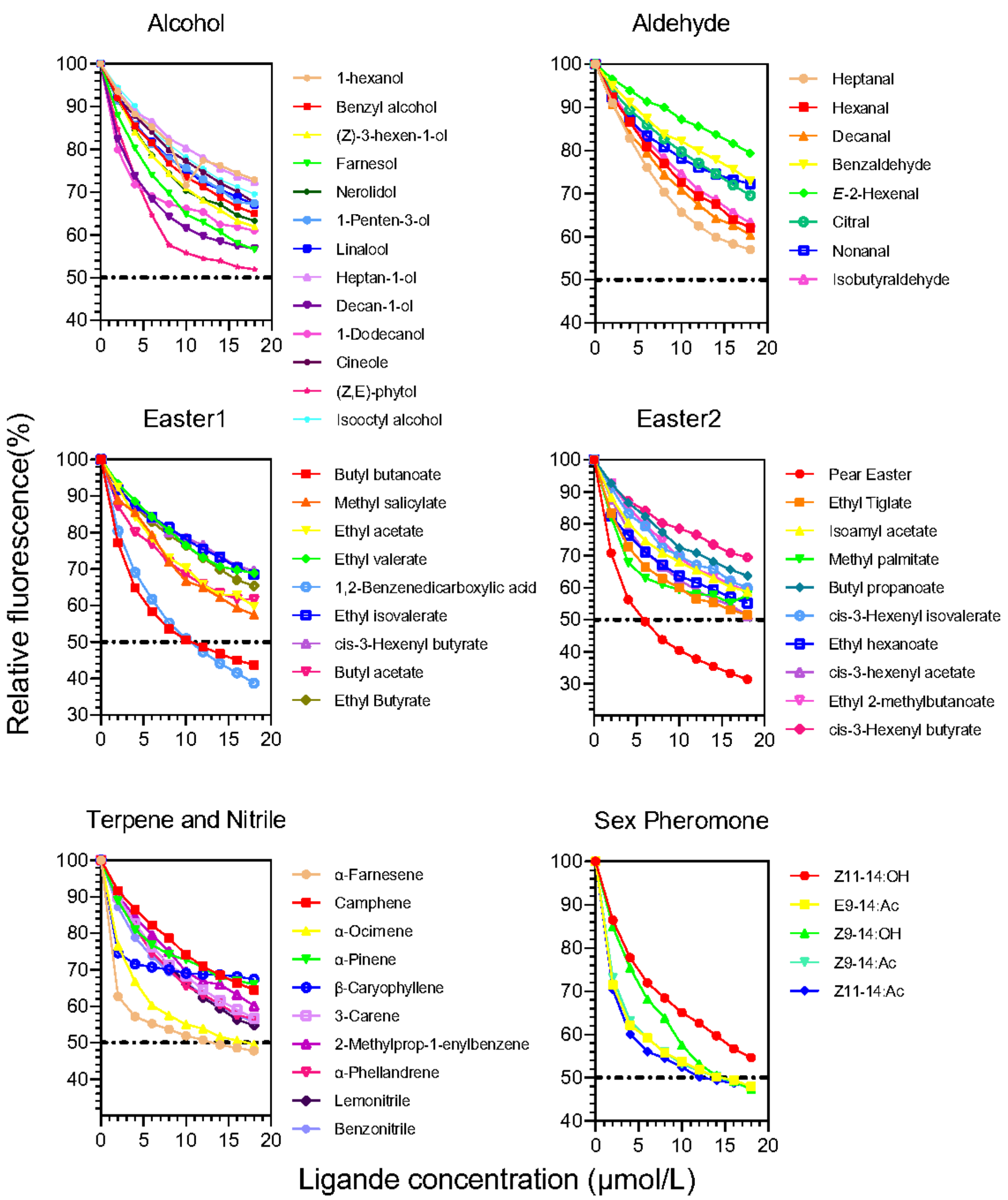

Figure S3. Competitive binding curves of AoraPBP3 to various ligands.

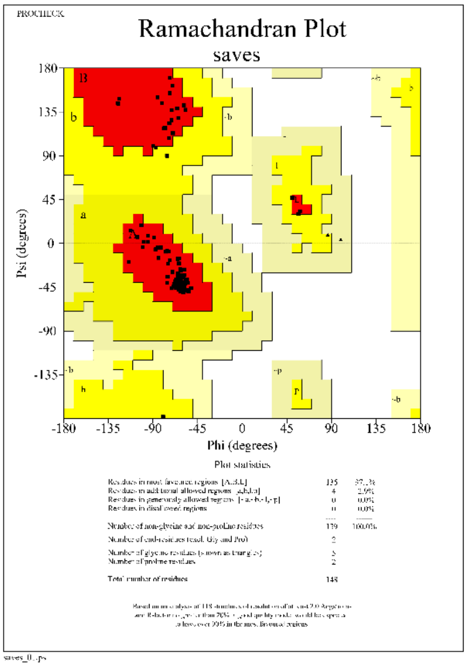

Figure S4. Ramachandran plots of AoraPBP3 model.

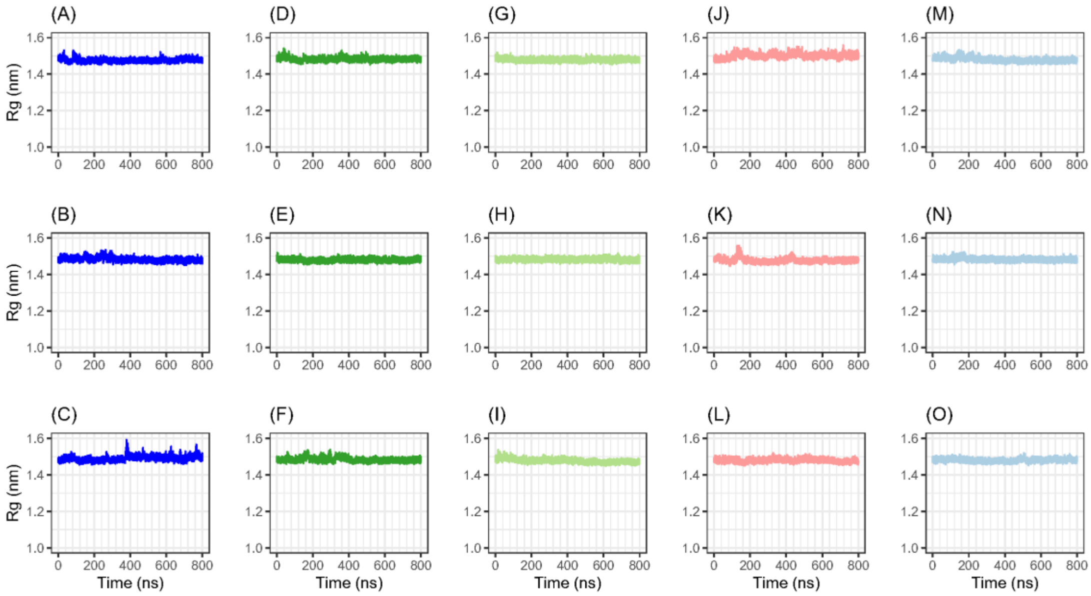

Figure S5. Radius of gyration (Rg) dynamics in the AoraPBP3-Ligand complexes. **(A-C)** AoraPBP3–*Z*9-14:Ac. **(D-F)** AoraPBP3–*Z*11-14:Ac. **(G-I)** AoraPBP3–*Z*9-14:OH. **(J-L)** AoraPBP3–*Z*11-14:OH. **(M-O)** AoraPPB3–*E*9-14:Ac.

Table S1. Primers used in this study.

Primer name	Sequence (5'-3')
**RT-PCR**	
qAoraPBP3-F	GCTGATCGATACCAGCGGAA
qAoraPBP3-R	AGAATCGCCACCAGCTTTGA
qAoraEF1-F	TGCCACACTGCTCACATT
qAoraEF1-R	GTCTCCGGACTTGATGGATTT
qAoraAct-F	GCCCTAGCTCCCTCTACCAT
qAoraAct-A	ACATCTGCTGGAAGGTGGAC
**Recombinant expression**	
eAoraPBP3-F	CGGGATCCGATACCATGATGGATCAGACG (BamHI)
eAoraPBP3-R	CCCAAGCTTGGGCTAGACATCAGTAAGGACTTCAG (HindIII)

Table S2. Amino acids within 5 Å around sex pheromone molecules based on molecular docking.

Ligand	Residues within 5 Å around the ligand
*Z*9–14:Ac	Leu14, Phe18, Phe39, Tyr42, Trp43, Ile58, Leu67, Ala79, Phe82, Ala83, Leu96, Leu100, Ile120, Phe124
*Z*11–14:Ac	Leu14, Thr15, Phe18, Leu19, Phe39, Tyr42, Trp43, Ile58, Val59, Ser62, Ile67, Leu100, Ala117, Ile120, Ala121, Phe124
*Z*9–14:OH	Leu14, Thr15, Ser16, Phe18, Leu19, Phe39, Tyr42, Trp43, Ile58, Leu67, Phe82, Leu100, Ala117, Ile120, Ala121, Lys122, Phe124
*Z*11–14:OH	Leu14, Thr15, Phe18, Leu19, Phe39, Tyr42, Trp43, Ile58, Met61, Leu67, Ala78, Phe82, Ala83, Leu100, Ala121, Phe124
*E*9–14:Ac	Met11, Leu14, Thr15, Phe18, Leu19, Phe39, Tyr42, Trp43, Ile58, Ser62, Leu67, Phe82, Leu100, Ala117, Ile120, Ala121, Phe124
